# Cecal volvulus caused by endometriosis in a young woman

**DOI:** 10.1186/s12893-015-0063-8

**Published:** 2015-06-24

**Authors:** Daisuke Ito, Susumu Kaneko, Kouji Morita, Shimizu Seiichiro, Masanori Teruya, Michio Kaminishi

**Affiliations:** Department of Gastrointestinal Surgery, Showa General Hospital, 8-1-1, Hanakoganei, Kodaira, Tokyo 187-8510 Japan; Department of Pathology, Showa General Hospital, Tokyo, Japan

**Keywords:** Cecal volvulus, Colonoscopy, Endometriosis, Laparoscopic ileocecal resection

## Abstract

**Background:**

Cecal volvulus is relatively rare. Moreover, to the best of our knowledge, a case of cecal volvulus caused by endometriosis has not yet been reported.

**Case presentation:**

A 41-year-old woman was admitted to our hospital with a 14-day history of subacute intermittent right lower quadrant abdominal pain. Simple abdominal radiography and abdominal computed tomography findings were suggestive of sigmoid volvulus, and she underwent an emergency colonoscopy. Following colonoscopic reduction, the patient’s symptoms resolved quickly, and elective laparoscopic surgery was scheduled 2 weeks after admission. Intraoperative examination revealed a significantly distended cecum and ascending colon, which was twisted around a short rope-like adhesion that connected the cecum and the mesentery of the transverse colon, whereas the sigmoid colon was neither twisted nor extended. We laparoscopically performed an ileocecal resection. The postsurgery histopathological examination revealed the presence of endometrial tissue in the short rope-like adhesion. This finding confirmed that cecal volvulus in this patient was caused by endometriosis.

**Conclusion:**

Cecal volvulus should be considered in relatively young women who present with atypical right lower abdominal pain. Whenever possible, secondary factors should be evaluated preoperatively, especially in relatively young patients.

## Background

Cecal volvulus involves the rotation of the cecum, terminal ileum, or ascending colon around its own mesenteric axis [1]. Incomplete intestinal rotation generally results in inadequate right colon fixation, which, in turn, can lead to the development of cecal volvulus. Other factors such as an anatomical susceptibility because of postoperative adhesion(s) can also cause volvulus. The only effective treatment for cecal volvulus is surgical intervention [2]. Surgery is usually performed as an emergency procedure, and laparoscopic techniques are rarely used [3]. Herein, we report a case of cecal volvulus due to endometriosis, which was treated with laparoscopic ileocecal resection.

## Case presentation

A 41-year-old woman with a 14-day history of subacute intermittent right lower quadrant abdominal pain was admitted to our hospital. No accessory symptoms were observed. She experienced similar symptoms approximately 5 years prior to this event. At that time, her symptoms resolved after fasting and the administration of intravenous fluids and antibiotics. Cecal volvulus was not noted.

**Fig. 1:**
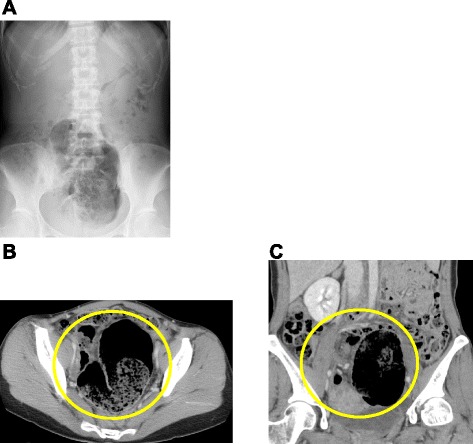
**a** Plain radiography showing a distended cecum without air-fluid levels. **b**, **c** Abdominal computed tomography showing dilated intestinal gas and a “whirl sign” in the middle of the abdomen

She did not have a history of any surgical procedures and any gynecologic diseases. She had a regular menstrual cycle and had not taken oral contraceptives or progestins. Her physical examination revealed a distended abdomen with localized tenderness in the right lower region without peritoneal signs. Laboratory tests reported a white blood cell count of 4820 cells/μL and C-reactive protein level of 0.06 mg/dL. Plain radiography showed dilated gas-filled loops of the colon. The colon was displaced in the pelvis but there was no evidence of air-fluid levels (Fig. 1a). Abdominal computed tomography showed both dilated intestinal gas like coffee beans in the pelvis and signs of twisting of the mesentery and mesenteric vessels (whirl sign), although these findings did not indicate which section of the large intestine was twisted (Fig. 1b, c).

**Fig. 2:**
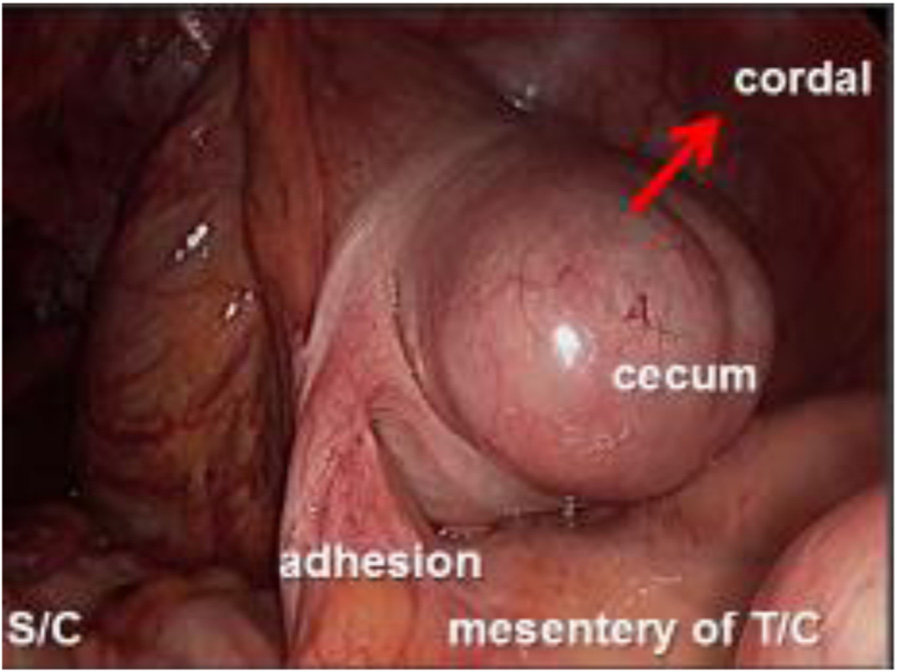
Intraoperative examination showing a distended cecum and the ascending colon twisted around a short rope-like adhesion connecting cecum and the mesentery of the transverse colon. S/C; Sigmoid colon, T/C; Transverse colon

**Fig. 3:**
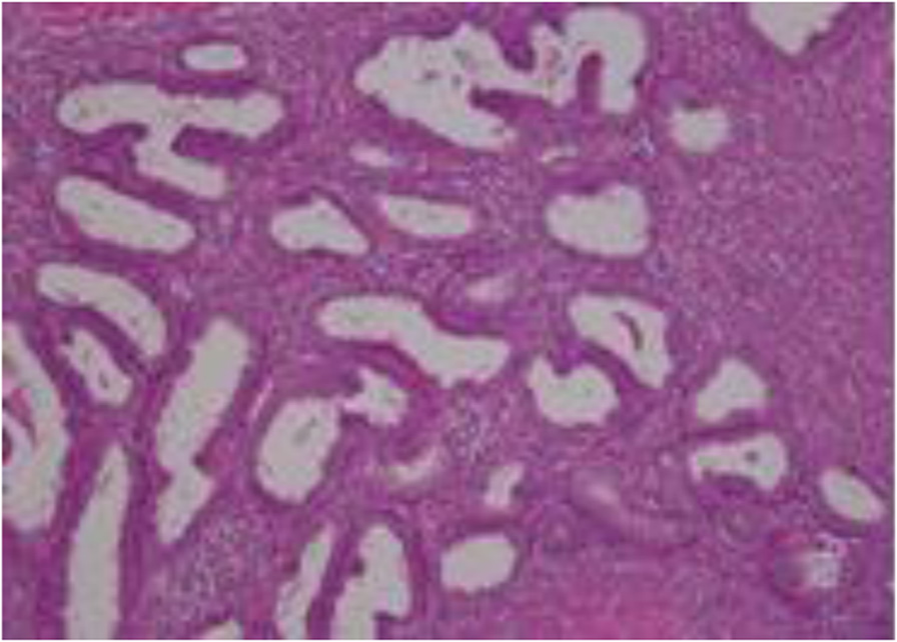
Histopathological examination showing a rope-liked adhesion caused by endometriosis (hematoxylin and eosin; 200× magnification)

We suspected sigmoid colon volvulus and performed an emergency colonoscopy. The large intestine was not ischemic and was filled with stool (Fig. 2). We successfully repositioned the colon, and the patient’s symptoms resolved soon after the colonoscopic reduction. We scheduled an elective laparoscopic sigmoid resection 14 days after the colonoscopy. Intraoperative exploration showed a significantly distended cecum and ascending colon, which was twisted around a short rope-like adhesion that connected the cecum and transverse colon mesentery. The intestinal wall showed no evidence of ischemia, necrosis, or perforation. Therefore, a laparoscopic ileocecal resection rather than a sigmoid resection was performed (Fig. 2). The postoperative course was uncomplicated. Histopathological examination revealed the presence of endometriosis in the short rope-like adhesion (Fig. 3). This finding confirmed a diagnosis of cecal volvulus caused by endometriosis. No other focus of endometriosis could be identified. The patient was discharged in good general condition 10 days after the laparoscopy. After the operation, she was diagnosed with endometriosis for the first time. The endometriosis was not found in the other organs on magnetic resonance imaging, and the patient has since been monitored without the need for any further treatment.

## Conclusions

The case presented here is unique for 2 reasons. Firstly, this was a relatively young woman who developed cecal volvulus due to endometriosis, and secondly, her cecal volvulus was resolved with emergency colonoscopy and elective laparoscopic ileocecal resection.

Cecal volvulus is a rare condition, which is associated with inadequate right colon fixation [4]. However, factors other than an anatomical susceptibility are thought to contribute to the development of this condition because the incidence rate of mobile cecum syndrome, a congenital abnormality in which the right colon does not properly fuse with the lateral peritoneum, is about 40 times higher than that of volvulus [1, 2]. These factors can be divided into 3 groups: (1) the presence of a support position (i.e., a postoperative adhesion or band) [5–7], (2) a change in the position of the colon (i.e., late-term pregnancy or jumping), and (3) colon stagnation (chronic constipation, high fiber intake, or psychiatric disorders) [8]. In the present case, cecal volvulus developed in a previously healthy female patient due to tissue adhesion from endometriosis. It is true that intestinal involvement occurs in 3–37 % of patients with endometriosis, and intestinal endometriosis typically takes the form of asymptomatic serosal implants that occasionally result in intestinal obstruction with recurrent abdominal pain [9]. However, to our knowledge, this is the first report of cecal volvulus due to endometriosis. Our finding indicates that secondary factors could cause cecal volvulus and that it is important to examine such factors, especially in relatively young patients who present with volvulus.

In general, the use of endoscopy in the diagnosis of acute cecal volvulus is limited because the success rate of colonoscopic reduction of cecal volvulus is only about 30 % [10]. The risk of colonic perforation and unsuccessful reduction is relatively high, and colonoscopic reduction is therefore not recommended as an initial treatment for cecal volvulus [3, 11]. Currently, surgical intervention is considered the most appropriate treatment option for the correction of intestinal obstruction due to acute cecal volvulus. However, we believe that flexible endoscopy would be indicated if a patient’s general condition is stable and the intestinal wall is not ischemic, necrosed, or perforated, as evidenced by enhanced computed tomography imaging findings. Endoscopic reduction of cecum volvulus can negate emergency surgery and it supports effective utilization of elective laparoscopic surgery. Laparoscopic surgery is less invasive, more economical, and has physical and cosmetic advantages, especially for relatively young female patients, such as the patient in this case.

Cecal volvulus should be considered in relatively young women who present with atypical right lower abdominal pain. Whenever possible, secondary factors should be evaluated preoperatively, especially in relatively young patients, so as to allow for appropriate treatment planning and to avoid unnecessary morbidity.

### Consent

Written informed consent was obtained from the patient for publication of this case report and any accompanying images. A copy of the written consent is available for review by the Editor of this journal.
